# Nitric Oxide: From Gastric Motility to Gastric Dysmotility

**DOI:** 10.3390/ijms22189990

**Published:** 2021-09-16

**Authors:** Eglantina Idrizaj, Chiara Traini, Maria Giuliana Vannucchi, Maria Caterina Baccari

**Affiliations:** 1 Section of Physiological Sciences, Department of Experimental and Clinical Medicine, University of Florence, 50134 Florence, Italy; 2 Research Unit of Histology and Embryology, Department of Experimental and Clinical Medicine, University of Florence, 50139 Florence, Italy; chiara.traini@unifi.it (C.T.); mariagiuliana.vannucchi@unifi.it (M.G.V.)

**Keywords:** gastric motility, nitric oxide, nitrergic neurotransmission

## Abstract

It is known that nitric oxide (NO) plays a key physiological role in the control of gastrointestinal (GI) motor phenomena. In this respect, NO is considered as the main non-adrenergic, non-cholinergic (NANC) inhibitory neurotransmitter responsible for smooth muscle relaxation. Moreover, many substances (including hormones) have been reported to modulate NO production leading to changes in motor responses, further underlying the importance of this molecule in the control of GI motility. An impaired NO production/release has indeed been reported to be implicated in some GI dysmotility. In this article we wanted to focus on the influence of NO on gastric motility by summarizing knowledge regarding its role in both physiological and pathological conditions. The main role of NO on regulating gastric smooth muscle motor responses, with particular reference to NO synthases expression and signaling pathways, is discussed. A deeper knowledge of nitrergic mechanisms is important for a better understanding of their involvement in gastric pathophysiological conditions of hypo- or hyper-motility states and for future therapeutic approaches. A possible role of substances which, by interfering with NO production, could prove useful in managing such motor disorders has been advanced.

## 1. Nitric Oxide

Nitric oxide (NO) is a ubiquitous gaseous molecule synthesized by almost all mammalian cells and exerts a variety of biological functions [1]. Since its discovery by Joseph Priestly in 1722 as a kind of colorless gas, NO was considered an environmental pollutant until 1980, when Furchgott and Zawadzki demonstrated that vascular relaxation induced by acetylcholine was dependent on the presence of endothelium and provided evidence for the release of a volatile humoral factor [2]. This substance, initially called endothelium-derived relaxing factor, is now recognized as NO. This discovery paved way for later recognition of NO synthesis pathways and its various biological functions. In fact, the importance of NO was established over years of research, demonstrating the central and peripheral role of NO in a wide variety of physiological and pathological conditions [3,4,5].

### 1.1. NO Biosynthesis

NO is synthesized from the amino acid L-arginine under the catalytic action of nitric oxide synthases (NOS), with L-citrulline as co-product. Three major NOS isoforms are usually expressed by different cell types: the constitutive isoforms, i.e., endothelial NOS (e-NOS or NOS III) and neuronal NOS (nNOS or NOS I), and the inducible NOS (iNOS or NOS II) [1]. The isoforms got their names based on their functions or the type of tissues in which they were firstly found [6]. All isoforms use three cofactors: flavin adenine dinucleotide (FAD), flavin mononucleotide (FMN), and (6R)5,6,7,8-tetrahydro-L-biopterin (BH4). The enzymatic activity of NOS is regulated by multiple factors, including substrate and cofactor bioavailability, calcium levels, protein levels, and dimerization as well as post-translational modifications [7].

NO produced by the constitutive nNOS isoform serves as a messenger in the central and peripheral nervous systems. In the central nervous system, nNOS is localized in the cerebellum and the brain. NO is involved in many functions, including neuronal plasticity, which helps in memory and learning processes [8].

Different NOS splice variants have been identified. In particular, the nNOS variants could be one membrane-associated nNOS (called nNOSα) or two soluble cytosolic nNOS (called nNOSβ and nNOSγ) [9]. The eNOS variants could be one full-length or two shorter variants. Up to 50% of enteric neurons of the mammalian gastrointestinal (GI) tract express the nNOS isoform, located either in the soma or the axons, which likely corresponds to the nNOSβ variant. The colonic muscle coat has reported to contain the highest number of nNOSβ-immunoreactive neurons compared with the other gut regions. Interestingly, all enteric neurons express full-length eNOS variant and iNOS with a limited distribution to the soma [9]. In turn, the smooth muscle cells (SMCs) express all of the NOS isoforms: in particular, the nNOS is located at the plasma membrane and likely corresponds to the nNOSα variant, while the full-length eNOS and the iNOS are cytoplasmatic. Moreover, nNOSα and full-length eNOS are also expressed by the gut pacemaker cells called interstitial cells of Cajal (ICCs) [9].

### 1.2. The NO-GC Pathway

The main receptor for NO is the NO-sensitive soluble guanylyl cyclase (NO-GC) [10], which is expressed in a variety of cell types [11] and signals through cGMP on to cGMP-dependent protein kinase (PKG), phosphodiesterases or, possibly, on to cGMP-regulated channels [12]. The receptor for NO is not G-protein coupled, but cytoplasmic and composed of α (α1, α2) and β (β1) subunits of sGC, α1β1 being the most common. Binding of NO to the N-terminal haem group of sGC causes a conformational change and generation of cGMP from the catalytic domain at the C-terminus [13].

Several cell types within the gut express NO-GC, including SMCs, ICC and telocytes (TCs) [14], also known as platelet-derived growth factor receptor alpha positive (PDGFRα+) cells (see the next section), all of which may contribute to the transduction of nitrergic signals [11]. ICCs and TCs/PDGFRα+ cells are indeed electrically coupled to SMCs, forming an electrical syncytium, known as the SIP (Smooth muscle cells, Interstitial cells of Cajal, TCs/PDGFRα+ cells) syncytium [15]. So, changes in conductance in any of the SIP cells can influence voltage-dependent processes in the other cells, thus neurotransmitter responses can be generated in any of the cells and conduct to other SIP cells. ICCs and TCs/PDGFRα+ are wound around varicose processes of enteric motor neurons and in close contact with varicosities of excitatory and inhibitory neurons, including those expressing nNOS. Thus, each SIP cell may be exposed to NO released from motor neurons, and there has been a significant effort to determine which cells mediate nitrergic responses [16]. However, even though the exact role of TCs/PDGFRα+ cells on NO signaling has not been elucidated yet, a strong expression of NO-GC in TCs/PDGFRα+ cells has been reported [17].

The increase in cGMP in response to NO is linked to several effectors in cells, including the cGMP-dependent protein kinase, PKG1, PDE and nucleotide-gated ion channels [18]. The expression and abundance of these effectors tend to define the mechanism of action of NO in specific types of cells. Post-junctional responses to NO are commonly attributed to PKG1 [19] even if the possibility that downstream effectors other than PKG1 contribute to nitrergic relaxation has been suggested [20]. On this view, it should be recognized that several PDEs are also expressed in post-junctional cells, including the dual substrate PDE3A that is expressed in ICCs and directly inhibited by cGMP via competition with cAMP for the active site. PDE3A could be a target for cGMP, and therefore, part of the downstream mechanisms causing the inhibitory effects of NO might be cAMP-dependent [16].

## 2. The Control of GI Motility

It is well known that GI motility is under myogenic, hormonal, and nervous control. The latter includes both the extrinsic innervation, represented by the two branches of the autonomic nervous system (i.e., the sympathetic and the parasympathetic), and the local intrinsic innervation, represented by the enteric nervous system (ENS). A characteristic feature of the GI tract is indeed to possess an intrinsic innervation (i.e., the ENS), described by Langley [21] as a third branch of the autonomic nervous system. The ENS includes the myenteric plexus (Auerbach plexus), localized between the circular and the longitudinal muscle layers, which is mainly involved in the motor control through its effects on SMCs and thereby regulating GI motility. Functionally, besides the afferent neurons and interneurons, motor neurons are indeed present in the ENS [22] which represent the final connection with SMCs of the circular and longitudinal layers. The ENS, due to its ability to receive local inputs, integrate information, generate outputs and elaborate reflexes even independently from the extrinsic innervation, gained the appellative of “little brain”. Nevertheless, both parasympathetic and sympathetic efferent fibers project to the enteric ganglia and may modulate the activity of the ENS, providing important integration, regulation, modulation and coordination of GI functions [23]. However, with particular reference to the physiological regulation of gastric motility, it has been shown that sympathetic nerves do not play an important role in such mechanism, which mainly occurs via the parasympathetic system, through the vagus nerve synapsing on the myenteric motor neurons [24]. The extrinsic parasympathetic efferent fibers may supply both excitatory and inhibitory inputs to the GI SMCs, since enteric neurons, according to the neurotransmitters they code for, are either excitatory or inhibitory motor neurons [25]. Notably, the enteric motor neurons may synapse, besides the SMCs, on other cell types within the gut such as the above-mentioned ICCs found within muscle bundles [26]. The existence of different subtypes of ICCs has been indeed reported, based on functional and morphological differences. For instance, in addition to the ICCs present in the myenteric plexus, having the specific property to generate slow waves and thus considered as pacemaker cells [27], there are also those localized within the longitudinal and circular smooth muscle layers and referred to as intramuscular ICCs which appear to be involved in enteric neurotransmission [28]. Moreover, TCs/PDGFRα+ cells have been recognized as a category of interstitial cells besides ICCs, that can be distinguished from the latter by their expression of PDGFR-α and their lack of cKit expression. Both TCs/PDGFRα+ cells and ICCs, probably form gap junctions with SMCs and they are found in close proximity to excitatory and inhibitory enteric motor neurons [14,16].

Growing evidence suggests that enteric glial cells too, participate in regulating different gut functions, including motility [29]. Enteric glial cells indeed express a variety of receptors for gut neurotransmitters and modulators, so that once glial cells are activated they modulate various gut functions [30,31].

### Role of NO in the Control of GI Motility

Enteric inhibitory motor neurons are mainly represented by the non-adrenergic, non-cholinergic (NANC) ones, whose nervous fibres release a variety of neurotransmitters. Among them, NO appears to be the main inhibitory neurotransmitter released from NANC fibres and responsible for GI relaxation [16].

Besides the different effect on various organs and tissues, NO seems indeed to have a crucial role in controlling GI motility and NO released by enteric nerves and/or produced by SMCs and ICCs acts as an inhibitory mediator, usually causing relaxation [17]. In this view, PKG appears to be the relevant effector for GI motility and phosphorylation of several target proteins is known to be part of NO-induced changes in gut motor responses [32].

Regulation of NO production in the range of physiological concentrations represents a key mechanism either to maintain its biological functions or to control its harmful effects. In this regard, the role of NO in physiological conditions has been well established in the motility of the different portions of the GI tract. Of note, studies in which knockout animal models have been used to focus on the functional effects of nitrergic signaling in the small intestine, reported NO/cGMP/PKGI as the main signaling pathway which influences GI smooth muscle tone [33]. On this view, mice lacking the β1 subunit of soluble guanylate cyclase (sGCβ1) fail to respond to NO donors or to NO released from enteric motor neurons, suggesting that the NO/cGMP signaling is of fundamental importance for the regulation of GI motility [17]. Moreover, the importance of NANC neural mechanisms involving nerves containing NO and tonic NO release in the regulation of cyclical fasting small intestinal motility has been reported in humans by Russo and collaborators since 1999 [34]. Furthermore, combined blockade of inhibitory neuromuscular transmission with L-N^G^-nitroarginine methyl ester (L-NAME) and apamin (blockers of NOS and SK channels, respectively) led to the disruption of peristalsis and irregular contractions of the circular muscle [17]. Interestingly, several studies demonstrated that NO donors could be able to disrupt the migrating motor complex [34,35]. Notably, topical NO donors have already been used in patients undergoing endoscopic retrograde cholangiopancreatography to relax the sphincter of Oddi and inhibit duodenal motility [36].

Several studies reported that the colonic contractile activity is suppressed by a basal release of NO since the inhibition of NOS leads to increased spontaneous contractions and/or tone [37,38]. NO has a significant role also on controlling colonic migrating motor complex (CMMC) movements. In this view, the NO-GC blockers as well as NOS inhibition cause an increase in the frequency of the CMMC, thus indicating that an endogenous NO release ordinarily acts to slow CMMC cycling [39] as the same effect on the CMMCs was also seen in the presence of tetrodotoxin [40].

A decrease of small bowel motility due to NOS II and III expression up-regulation induced by the hormone relaxin, which attains high circulating levels during pregnancy, has been reported [41]. Such NO-mediated effect may be regarded as a physiological mechanism activated during pregnancy, likely addressed to increase the intestinal transit time so enhancing the opportunity for absorption of nutrients to fulfill the fuel needs of the mother and the growing fetus.

Changes in NOS isoforms expression, which often represents a shared target for many substances to modulate the amount of NO production, have been revealed both in physiological or pathophysiological conditions [42]. Notably, although all the three different NOS isoforms and their splice variants are expressed in GI cells, the nNOS isoform producing NO as neurotransmitter plays the most important role in motility. In fact, deficiency of neurons that express NOS has been reported to be associated with numerous gut motor diseases in different pathological conditions leading to an impairment of GI motility [17]. It is interesting to note that changes, in addition to those related to the number of enteric neurons containing NOS, were observed in either NO release or cellular and subcellular of different NOS isoforms expression and associated with various GI diseases [42].

Moreover, many substances, including hormones, have been reported to exert a modulatory action on the nitrergic neurotransmission further underlying the importance of this innervation in the control of GI motility [43,44,45,46,47,48,49].

Notably, an altered NO production/release (either defective or excessive) has been indeed reported to be responsible for some GI motor dysfunctions [16,42]. In fact, different pathological motor conditions are related with a defective or excessive NO production in the different portions of the GI tract of both humans and animals [17,50,51]. On this view, when the small bowel motility is severely impaired, patients may present a picture of chronic intestinal pseudo-obstruction or even constipation which is thought to be related to the enteric NO system [52]. Whereas an enhanced NO release from NANC fibres in colonic dysmotility of patients with slow-transit constipation has been observed [53], the abolition of the colonic nitrergic innervation has been reported in patients with other pathological conditions [17]. Furthermore, in active ulcerative colitis and Crohn’s disease elevated activity of NOS are reported with an enhanced generation of NO found in colonic mucosal biopsies [42]. A toxic megacolon in patients with ulcerative colitis could be caused by overproduction of NO by iNOS in the colonic smooth muscles and selective iNOS inhibition has been suggested as a treatment strategy in this life-threatening condition [54]. As well, increased NOS-IR in the myenteric plexus has been reported to be involved in colonic hypomotility observed in chronic pancreatitis induced in rats by trinitrobenzene sulfonic acid (TNBS) infusion, thus suggesting NOS contribution to the pathogenesis of colonic dysmotility [55]. Therefore, upregulation inhibition of NOS-IR neurons by some potential drugs has been suggested as a pharmacotherapeutic tool also for improving colonic hypomotility associated with chronic pancreatitis.

**Figure 1:**
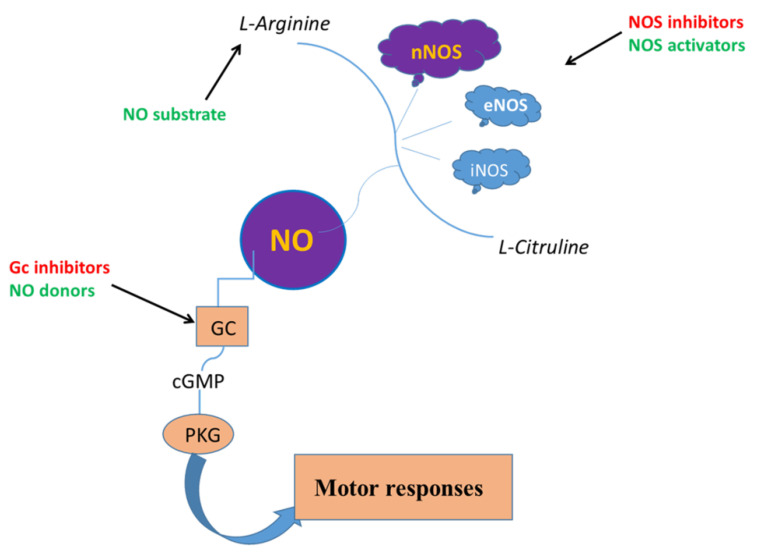
Simplified scheme representing the main signaling pathway through which NO influences the motor responses in the gastrointestinal tract and some possible targets for therapeutic intervention. Enhancement of NO signaling can be achieved by NO donors, by providing excess arginine substrate or enhancing signaling pathways that activate NOS functions (green color). NO signaling prevention may be achieved by guanylate cyclase (GC) function or NOS activity inhibitors (red color).

Thus, NO is involved in both physiological and pathological conditions thus a good operation of the NO system and NOS expression is important for a proper functioning of the gut smooth muscle. A schematic illustration of the main pathway through which NO controls GI motility and some of the substances able to modulate its effects is reported in Figure 1.

However, it should be taken into account that the modulation of NO production is a complex mechanism complicated by the fact that there are a variety of stimuli of different nature (e.g., dietary component, inflammation, bacteria) that may also affect NOS expression [4,8,42,56,57,58,59]. Thus, some aspects on central and peripheral role of NO in health and disease that remained unsolved could be elucidated by further integrated studies, including those on gut neuro-immune interactions and bi-directional microbiota-gut-brain axis.

## 3. NO in Gastric Motility and Dysmotility

Although anatomically in the stomach different regions can be distinguished (i.e., cardia, fundus, corpus, antrum and the pyloric sphincter), from a functional point of view the organ may be divided into two main portions, namely the proximal and the distal stomach: the former (which includes the fundus and the first third of the corpus) having the main function of reservoir aimed to receive the contents from the esophagus and to store it temporarily; the distal one (which includes the remaining two-thirds of the corpus and the antro-pyloric region) being mainly deputy to gastric emptying, other than mixing of the contents. In agreement with their physiological functions, different motor responses occur in the two regions. For instance, in the proximal stomach, particularly in the fundus, tonic contraction/relaxation prevails and spontaneous rhythmic contractions are absent, since pacemaker ICCs in the myenteric plexus are lacking. At variance, rhythmic contractions are present in the more distal portions of the stomach.

### 3.1. NO in Gastric Motility

NO appears to play an important role particularly in the proximal stomach. Of note, NO has been reported to mediate the accommodation reflex, which occurs in response to gastric filling and resulting in a decreased tone of the proximal gastric region [60,61].

It is known that the tone of the gastric proximal portion decreases during food intake. This process of active relaxation is mediated by different parasympathetic reflex pathways, which lead to an increased NO release in humans [62]. Even if accommodation is usually considered as a mechanism which allows the stomach to prevent a rise in intragastric pressure during food intake [63], studies in humans have reported that ingestion of nutrients evokes an initial drop in the intragastric pressure, followed by a gradual recovery [64]. Gastric accommodation is based on fundal relaxation and studies in humans have shown such relaxation to be dependent upon NO [65,66]. In keeping, the ability of NO to cause fundal relaxation has been widely reported in both humans and animals, and NO synthesis inhibitors as well as the soluble guanylate cyclase inhibitor, 1H-[1,2,4] oxadiazolo [4,3-a]quinoxalin-1-one (ODQ), have been shown to counteract the relaxant responses elicited by chemical or electrical stimulation of nitrergic neurons [45,61,66,67,68,69,70,71]. In this view, we observed that either tetrodotoxin or the NO synthesis inhibitor L-N^G^-nitro-arginine (L-NNA) abolished the relaxant responses evoked, in NANC condition, by electrical field stimulation in strips from the mouse gastric fundus, thus indicating their nervous and nitrergic nature [45]. Moreover, studies conducted in fundal strips from knockout mouse lines for NO-GC or the downstream target PKGI, reported the abolition of NO-induced fundal relaxation in GCKO mice and an impaired response to either NO donors or the cGMP analogue (8-Br-cGMP) further highlighting the importance of NO/cGMP-mediated signaling in gastric fundus [19].

In the proximal stomach, a variety of substances, including hormones, have been reported to exert a neuromodulatory role on the nitrergic transmission in rodent whole organ as well as in gastric fundal strips [43,44,45,46]. For instance, in these latter preparations, it has been reported that both the hormones glucagon-like peptide-2 (GLP-2) and relaxin, by up-regulating NOS I and NOS I plus NOS III expression, respectively, exert a modulatory action on the nitrergic neurotransmission. As a result, a decrease in amplitude of the neurally-induced contractile responses and an increase of the relaxant ones were observed in the mechanical experiments [43,45,72]. The depression of the contractile responses caused by an increased NO release during electrical field stimulation is in keeping with the following consideration: gastric motor responses represent a balance between nervous excitatory (mainly cholinergic) and inhibitory (NANC) influences both exerted, during electrical stimulation, on the smooth muscle.

Interestingly, it has been reported that adipocyte-released peptides too are involved in the control of gastric motility by influencing NO production/release [73]. In this view, adiponectin has been recently shown to modulate the nitrergic neurotransmission and to induce NO-mediated relaxant effects in gastric strips from mice [44], through the activation of the AMPK pathway and the involvement of glial cells of the myenteric plexus [74]. The ability of adiponectin to cause gastric relaxation led to hypothesize that this effect may represent an additional peripheral mechanism engaged by the hormone to reinforce its central anorexigenic effects [75]. In this regard, many substances that centrally modulate food intake also influence the GI motor phenomena, which represent a source of peripheral signals involved in the control of feeding behavior through the gut-brain axis [76,77]. Particularly, motor changes related to the stomach are recognized as key mediators of the hunger-satiety cycle. Both gastric accommodation and emptying indeed play a key role in the regulation of stomach distension [78]. The latter triggers stretch and tension, so stimulating mechanosensitive receptors which, in turn, communicate their information through the activation of the vagal afferent nervous fibers. These latter send signals to the hypothalamic regions involved in the regulation of food intake, through the interposition of the nucleus of the tractus solitarius [63]. Thus, besides anorexigenic gut hormones, such as cholecystokinin and glucagon-like peptide-1, which cause gastric distension and slow down emptying, satiety induced by gastric distension can also be regulated by some adipocyte-released peptides [63,73]. So, NO being the major responsible for gastric relaxation might also have a role in the peripheral regulation of the hunger-satiety cycle. Interestingly, it has been reported that stimulation of NO-GC, found to be expressed in both white and brown fat cells [11], induces ‘browning’ of white adipocytes and enhances the differentiation of human brown fat cells [79]. Taken together, the above observations suggest that NO-GC by modulating energy expenditure and contributing to satiety signals, through gastric relaxation, could also peripherally regulate the energy balance.

Despite the physiological role of NO reported in the proximal region of the stomach, little information is available in the literature on its role on the motility of the distal portion (corpus or antrum), where NO does not appear to exert a key role in keeping with the low density of nitrergic neurons in this region [80]. Nevertheless, NO has been reported to be involved in the regulation of gastric emptying in humans, in which exogenous NO has been reported to inhibit gastric emptying and antral motor activity [81]. This observation is in keeping with the above-described effects of NO in the proximal stomach aimed at inducing satiety. In agreement, evidence exists that a delayed gastric emptying is related to an increase in the feeling of satiety, which leads to stop introducing food and that obese subjects present enhanced gastric emptying [82]. Moreover, an inverse correlation between satiety and gastric emptying has been reported in healthy humans [83].

Even if NO has been reported to inhibit gastric emptying in humans, it has been also shown to relax the gastric pyloric sphincter [84], where nitrergic nerves have been revealed in high density [85], thus playing a fundamental role on regulating the transit of chyme from the stomach into the duodenum. In this view, it has been reported that NOS inhibitors delay gastric emptying in several species [86,87]. Recent advances in the understanding of the physiological circuits regulating gastric emptying have been done, further supporting the involvement of inhibitory neurons in the myenteric plexus that act by releasing NO [24].

### 3.2. NO in Gastric Dysmotility

An impaired NO release/production has been reported to be implicated in some gastric motor dysfunctions, in accordance with a NO role as the main NANC inhibitory neurotransmitter. In this view, mice deficient in nNOS, NO-GC or PKGI develop a hypertrophic pylorus and an enlarged stomach which result in reduced gastric emptying [88]. In humans a reduction in nNOS RNA and protein expression may account for infantile hypertrophic pyloric stenosis [89]. Thus, NO-GC and PKGI could be of essential importance in functional nitrergic neurotransmission leading to gastric emptying [17]. Of note, knockout mice lines generated by the deletion of exon 2 which lacks only in the nNOSβ splice variant of nNOS isoform (the alternative splice variants still remain expressed in many regions of the GI tract) showed an enlarged stomach and hypertrophy of the pyloric sphincter [90]. In a second nNOS knockout line generated by the deletion of exon 6, which intend lacks in all nNOS isoforms, the animals showed a more severe gastrointestinal phenotype as liquid diet was required for survival after weaning [91].

GI motor dysfunctions have been also reported in patients with Duchenne muscular dystrophy [92]. Experiments carried out on *ex vivo* gastric preparations from mice with muscular dystrophy, due to mutated dystrophin gene (mdx mice), have suggested a defective production/release of NO: upregulation of nNOS expression by the hormone relaxin has been indeed reported to counteract the altered contractile and relaxant responses in the gastric fundus of mdx mice [93].

Of note, gastroparesis (gastric stasis), a clinical condition resulting from delayed gastric emptying with no apparent obstruction, is accompanied by GI symptoms and usually associated with diabetes or considered idiopathic. Several hypotheses have been advanced to explain gastroparesis pathophysiology, which is still poorly understood and not well defined. However, the main mechanism associated appears to be the loss of both nNOS expression and ICCs [94]. In keeping, loss of nNOS in animals has been related to diabetic GI diseases [95] and changes in nitrergic neurons and ICCs have been described in patients with either idiopathic or diabetic gastroparesis [96,97,98]. Loss of ICCs in mice has been suggested to be a key factor in the development of delayed gastric emptying [99]. On the other hand, the consequences of ICCs loss on the conduction velocity of gastric slow waves have not been defined yet and, unexpectedly, the loss of ICCs has been observed to be associated with a higher slow wave speed in the human stomach [100]. As reported by the same authors, the increase in slow-wave velocity can modulate gastric emptying higher, even if in gastroparesis other pathological factors should dominate to prevent emptying in humans [100]. Anyway, the observation that degeneration or loss of ICC is involved in different GI dysmotilities underlines the importance of ICC regarding both pacemaking and neurotransmission [101]. In this view, gastroparesis also involves impaired fundal accommodation, abnormal small bowel transit, and delayed colonic transit and studies are in progress to determine the pathophysiology and the development of new therapeutic approaches [102,103]. In this perspective, help can also come from studies carried out on animal models. In this view, the observation that in mice expressing a heme-deficient NO-GC (apo-sGC mouse) gastric emptying was delayed, suggests apo-sGC mice as a valid model to investigate motility diseases such as gastroparesis [104]. Interestingly, it has been recently reported that differentiated PDGFRα+ cells may represent a novel *in vitro* model to conduct functional studies of NOS and to identify new therapeutic targets for treatment of diabetic gastroparesis [105].

## 4. NO and Possible Therapeutic Strategies

Modulation of NO production has been suggested as a possible therapeutic strategy in those motor diseases ascribable to defective or excessive NO production. In this view, physiological compounds, including hormones, able to interfere with NO synthesis, by acting on the specific NOS isoforms expression, or NO-donor drugs can represent useful tools to prevent or treat motor dysfunctions. The importance of this topic is underlined by the emerging researches searching for natural or chemical compounds targeting NO [106,107]. However, it should be remembered that most of the data relating to the effects of NO on GI motor responses derive from studies carried out in animals and caution is mandatory when transferring them to humans.

Animal studies have indeed undoubtedly expanded our understanding of NO effects on GI motility. Particularly, the use of genetically modified animals has provided and will continue to provide important information on the topic. For instance, genetically modified strains of mice lacking NOS or NO-GC and target-free strains downstream of the NO/cGMP cascade such as PKGI [32,108] have proved useful models for elucidating the effects of NO on GI motility. However, as reported by Groenberg [17], notwithstanding similarities in smooth muscle morphology and NO/cGMP signaling between humans and mice, the nitrergic pathways may not necessarily be identical. Even animal species-related differences with respect to NO-GC expression in the small intestine have been reported between guinea pigs and mice [109]. This is one more reason to be careful when translating results from animals to humans, especially for the development of new therapeutic strategies using NO. On the other hand, the importance of the regulation of NO production by eNOS in the control of vascular smooth muscle tone is widely recognized [5]. Drugs that generate NO, known as nitrovasodilators, have long been used to reduce blood pressure (and other coronary artery diseases) and treat angina pectoris and a new class of NO donating nonsteroidal anti-inflammatory compounds (NO-NSAIDs) has been developed (see [1]). These NO-NSAID, anti-inflammatories combined with precursors of the mediator or with inhibitors of the iNOS, are evaluated for GI injury treatment [56]. NSAID-gastroenteropathy, inflammatory bowel disease (IBD) and irritable bowel syndrome (IBS) are among the most common disorders affecting the GI tract. NO has been demonstrated to contribute to the pathogenesis of such disorders and for each case, NO or an inhibitor of NO synthesis has been proposed as a treatment. Thus far, however, NO-based therapies for these disorders have not been successfully translated to the clinic or the market [110].

However, due to the major role of NO in the control of GI motility, it could be hypothesized that also substances which indirectly modulate NO production may represent a possible therapeutic strategy in the treatment of gut motor dysfunctions. For instance, Otilonium bromide (OB), a drug with spasmolytic activity and extensively used to treat patients affected by IBS [111], has been reported also to increase nNOS expression [112], thus increasing NO production.

Furthermore, drugs which are not specifically marketed to counteract GI motor disorders but which influence NO production, could be seen as potential therapeutic agents for this purpose. This is the case of liraglutide, a glucagon-like peptide-1 receptor agonist, which is employed to reduce hyperglycemia and to control food intake [113]. Liraglutide has been indeed recently reported, other than to ameliorate the function of the ENS, leading to the normalization of colonic function in people with type 1 diabetes [114], to improve NOS activity and increase NO production [115].

## 5. Conclusions

All the above evidence suggests that NO plays a key role in the motility of the GI tract both in physiological and pathological conditions. Particularly, NO has been reported to be involved in the two main physiological functions of the stomach, namely accommodation and emptying, thus controlling the motor phenomena of either the proximal or the more distal portions of the organ. Going a step further, a link between the effects of NO on gastric motility and the regulation of food intake can be speculated.

**Figure 2:**
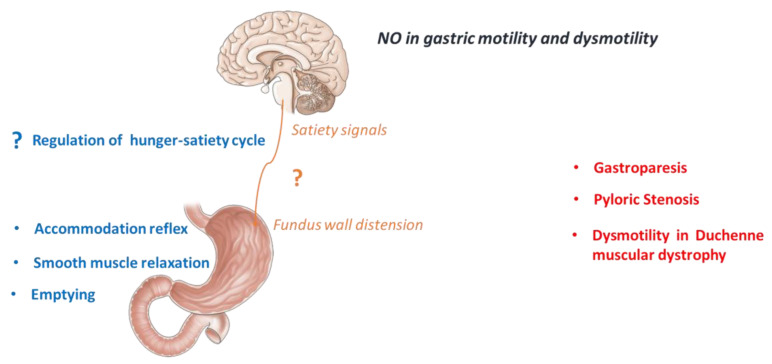
Physiological role of NO in the control of gastric motility (in blue) and the main motor dysfunctions of the stomach associated to a NO system alteration (in red) reported in humans or animals.

Moreover, an altered NO production/release has been reported to occur in some pathological conditions related to motor dysfunctions. A simplified schematic representation of the role of NO in physiological and pathological conditions related to gastric motility is reported in Figure 2.

However, notwithstanding the great progresses that have been made in the knowledge of NO biosynthesis and its signaling pathways, the use of compounds capable of targeting NO in the clinical approach of gastric hypomotility or hypermotility states certainly deserves to be more deeply investigated.

## Data Availability

Not applicable.
